# Primary Posterior Neck Hydatid Cyst: A Case Report and Review of the Literature

**DOI:** 10.1155/crot/6909432

**Published:** 2025-09-26

**Authors:** Gibran Atwi, Charbel Saad, Samer Serhal, Rami Saade

**Affiliations:** Department of Otolaryngology Head and Neck Surgery, Lebanese American University Medical Center-Rizk Hospital, Beirut, Lebanon

**Keywords:** *Echinococcus granulosus*, hydatid cyst, neck cyst, pediatric hydatidosis, zoonotic infection

## Abstract

**Introduction:** Hydatid cyst disease is an endemic parasitic infection caused by *Echinococcus granulosus*. Hydatid cysts occur mainly in the liver and lungs and are rare in the head and neck region, even in endemic areas. Due to their nonspecific clinical manifestations, diagnostic challenges are encountered in such atypical presentations.

**Case Presentation:** We report the case of a 6-year-old girl who presented with a progressively enlarging, painless left postauricular mass. Radiological assessment revealed a well-circumscribed, unilocular cystic lesion at level V of the neck. Surgical resection was performed, and histopathology confirmed a hydatid cyst. Postoperatively, the patient was treated with albendazole. No recurrence was observed at follow-up.

**Clinical Discussion:** Hydatid cysts in the cervical region are exceptionally rare, with only a few cases documented in the literature. The disease can remain asymptomatic for years, often presenting as a slow-growing mass. Imaging with ultrasound and CT is critical in preoperative diagnosis. The gold standard of treatment remains complete surgical excision, reinforced by anthelmintic therapy to prevent recurrence.

**Conclusion:** Although rare, hydatid cyst disease should remain among the differential diagnoses of cystic neck masses, especially in endemic areas. Early diagnosis and intervention are essential in evading complications of the disease such as anaphylaxis or recurrence. Ultimately, prevention remains the most effective strategy, emphasizing the need for community awareness, hygienic practices, and coordinated control efforts to break the parasite's life cycle.

## 1. Introduction

Hydatid disease is a zoonotic parasitic infection, caused by the larval stage of *Echinococcus granulosus* [1]. While the liver and the lungs are the most commonly involved sites, primary cervical hydatid cysts are exceedingly rare, accounting for less than 2% of all reported cases of hydatid disease [2–6]. Given the rarity of this location, these are frequently misdiagnosed, often leading to delays in appropriate management.

Here, we present a very rare occurrence of an isolated hydatid cyst in the posterior neck of a pediatric patient. This case report highlights the diagnostic challenges, management considerations, and the importance of including hydatid disease in the differential diagnosis of cystic neck masses, especially in endemic regions.

This work has been reported in accordance with the SCARE 2020 Guidelines [7].

## 2. Case Presentation

**Figure 1:**
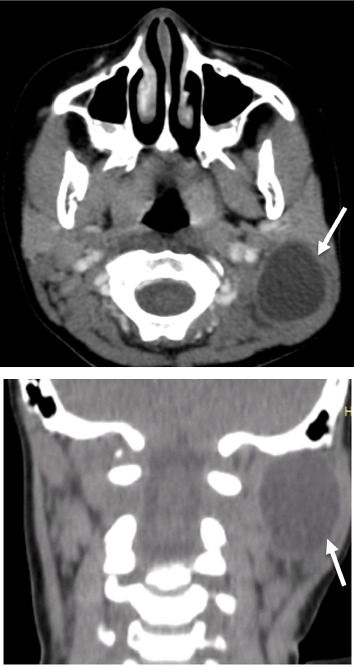
Preoperative axial and coronal CT scan images of the neck showing a left posterior neck unilocular cystic lesion (arrow).

A 6-year-old girl presented to the ENT clinic for 3-month history of a slowly growing left postauricular cervical mass. The patient's family denied any history of fever, pain, dysphagia, odynophagia, or weight loss. The girl resides in a rural area of Lebanon, where livestock farming is common and sanitation infrastructure is limited. Her family is of low socioeconomic status and lives in close proximity to domesticated animals, including sheep and dogs. The physical exam revealed an oval-shaped, soft, nontender mass. The overlying skin showed no signs of inflammation, and no cervical lymphadenopathy was detected. Routine laboratory testing was unremarkable. A neck ultrasound was performed next, and it showed a well-circumscribed anechoic cystic mass measuring 34.7 × 28.6 mm in the left postauricular area. No cervical adenopathy was identified. A contrast-enhanced computed tomography (CT) scan was subsequently performed to better delineate the anatomic relationships of the lesion, assess any soft tissue component or calcifications, and evaluate for potential invasion or compression of adjacent structures. It showed a level V unilocular cystic neck mass. It was seen posterior to the parotid gland and to sternocleidomastoid muscle, anterior to trapezius muscle, and independent from the carotid space. The mass was not enhancing, very well circumscribed, with no soft tissue component. While MRI is superior to CT in soft tissue characterization and avoids radiation, CT was chosen due to faster accessibility and high-resolution depiction of bone and soft tissue anatomy, offering rapid, detailed visualization of the lesion's extent and relation to vital structures. The mass looked benign, but it has been present for some time, slowly growing, and so the decision was taken to have it removed (Figure 1).

**Figure 2:**
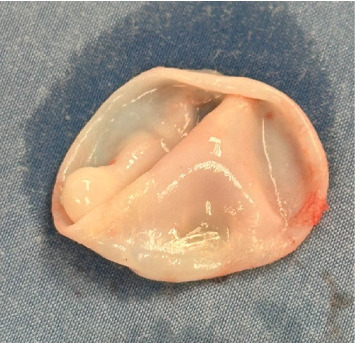
Excised hydatid cyst material.

**Figure 3:**
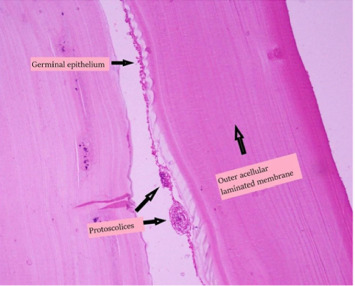
Photomicrograph of hematoxylin and eosin preparations of the specimen demonstrating acellular laminated eosinophilic structure of the hydatid cyst.

Complete resection of the cyst including tissues surrounding the cyst was performed. During the procedure, the cyst partially ruptured, and it was found to contain clear fluid and surrounded by a white germinal membrane. Since the lesion did not raise oncologic concern, and given the characteristic features seen intraoperatively, which raised the suspicion for hydatid disease, a frozen section was not deemed necessary. The mass was sent for final pathology, which revealed an echinococcal cyst (hydatid cyst) (Figures 2 and 3).

Further workup was done, including a chest radiograph and a liver ultrasound, both of which were unremarkable. The postoperative course was uneventful. The patient was referred to an infectious disease specialist and was started on albendazole 400 mg/day for 3 months, due to intraoperative rupture of the cyst, which increases the risk of microscopic spillage and recurrence. Prolonged therapy in such cases has been recommended, despite the known risk of hepatotoxicity. Liver function was monitored regularly, and the patient remained stable throughout the treatment. At 3-month follow-up, the patient remained disease free.

## 3. Discussion

Hydatid disease, caused by the larval stage of *Echinococcus granulosus*, is a significant parasitic infection with a global burden, particularly in livestock-rearing areas such as the Middle East, North Africa, South America, and Central Asia [1]. Humans become accidental hosts when they ingest eggs from contaminated food, water, or direct contact with infected animals. The larvae penetrate the human body through the intestinal mucosa. They enter the bloodstream and primarily lodge in the liver (65%–75%) and lungs (15%–25%), as these organs serve as initial filters [8, 9]. However, in rare cases, hydatid cysts bypass these barriers and disseminate to other organs, including the spleen, kidneys, heart, bones, brain, and soft tissues such as the head and neck region [10–15].

Hydatid disease, while endemic in several regions worldwide, is rarely encountered in the head and neck region. Mechanisms for spread remain unclear; however, it may be likely hematogenous or lymphatic [16]. Given the rarity of hydatid cysts in this location, they are frequently misdiagnosed as benign cystic lesions, such as branchial cleft cysts, thyroglossal duct cysts, or lymphangiomas [17–20]. In addition, its asymptomatic insidious presentation can make diagnosis particularly challenging.

Initial assessment of hydatid cysts is mainly through ultrasonography (USG) and CT, with definitive diagnosis relying on histopathological confirmation.

The World Health Organization Informal Working Group on Echinococcosis (WHO-IWGE) developed in 2001 a classification to describe hydatid cysts, primarily based on imaging, and reflects the stage of cyst development and viability and helps guide treatment decisions. The classification is summarized in Table 1 [21, 22]. In our case, imaging showed a well-defined, unilocular anechoic lesion without internal septations or calcifications, which is consistent with a Type I cyst. This is typically considered an early, active stage of hydatid disease.

Serologic testing (e.g., ELISA for *Echinococcus* antibodies) can support the diagnosis of hydatid disease. However, serology may have variable sensitivity depending on cyst location, with lower detection rates in extrahepatic or unusual sites such as the head and neck [9]. In this case, serology was not initially performed due to the unexpected nature of the diagnosis. Following the histopathologic confirmation, further imaging of the chest and abdomen was conducted to rule out systemic involvement, both of which were negative.

These cervical cysts are frequently confused with other more common cysts in the cervical region, such as congenital malformations, metastatic cystic lymphadenopathy, or benign tumors. Cystic lymphangiomas, which arise due to the failure of primitive lymphatic sacs to connect with the venous system properly, are one of the most frequently observed congenital lesions. These lesions rank among the most common congenital cystic masses in children, accounting for around 6% of the benign childhood lesions, typically detected by the age of 2 years, coinciding with peak lymphatic growth. They appear as soft, compressible masses and can remain asymptomatic but may rapidly enlarge due to hemorrhage, secondary infection, or trauma. These masses tend to spread into spaces and surround nearby anatomical structures. On USG, they usually appear as multiloculated, thin-walled cystic structures with minimal or absent Doppler flow. On CT and MRI, they present as poorly defined, multiloculated, anechogenic lesions with thin walls, and no enhancement, though they may show increased attenuation or echogenic content if associated with bleeding or infection.

A third branchial arch cyst, which represents remnants of embryonic branchial apparatus, is also seen in the posterior cervical triangle. Generally, these are painless, unilocular structures and unlike hydatid cysts, branchial cleft cysts lack internal septations and daughter cysts.

In contrast to congenital lesions, metastatic lymphadenopathy—particularly from papillary thyroid carcinoma (PTC)—is a potential malignant etiology for cystic cervical masses, predominantly affecting adolescents and young adults. PTC frequently spreads via the lymphatic system, and can manifest with cystic lymph node metastases. These nodes often exhibit vascularized septa and may contain calcifications or colloid material, and can be relatively easy to distinguish from hydatid cysts on USG. Even though fine-needle aspiration (FNA) is a standard diagnostic procedure for cystic neck masses, this procedure is discouraged in suspected hydatid disease due to the potential for parasitic spread, rupture, and anaphylactic reactions. The identification of a unilocular cyst without vascularized septa should prompt consideration of a hydatid cyst, particularly in endemic regions.  Table 2 summarizes the different differential diagnoses to be considered when a patient presents with a posterior cystic neck mass.

In pediatric cases, hydatid disease exhibits distinct characteristics compared with adult presentations. First, children are more likely to develop pulmonary rather than hepatic involvement. This is probably due to a more fenestrated hepatic filter that allows parasitic larvae to bypass the liver and reach the lungs. Despite these differences, head and neck involvement remains exceptionally rare in both children and adults [23, 24].

Surgical excision remains the treatment of choice, with special precautions taken to avoid intraoperative spillage and secondary dissemination. Adjunctive medical therapy with albendazole is recommended postoperatively to prevent recurrence [22].

The Puncture–Aspiration–Injection–Reaspiration (PAIR) technique is another minimally invasive approach used primarily for hepatic hydatid cysts, particularly those in types I and II according to the WHO classification [21, 22]. However, it is not generally recommended for cysts in the head and neck region due to proximity to critical neurovascular structures, risk of anaphylaxis, and challenges in ensuring complete evacuation. In this case, hydatid disease was not suspected preoperatively and the lesion was surgically accessible, well circumscribed, and excision offered a definitive cure with pathology confirmation.

A key intraoperative challenge was the rupture of the cyst, significantly increasing the risk of anaphylaxis and secondary dissemination. Postoperative prolonged albendazole therapy is crucial in preventing recurrence. Administered at a dose of 10–15 mg/kg/day over a minimum of three months, albendazole plays a critical role in reducing the viability of residual parasitic elements and minimizing disease dissemination [25].

Ultimately, prevention remains the most effective strategy to break the parasite's life cycle. Effective control requires a One Health approach, integrating human, animal, and environmental health sectors. Key strategies include regular deworming of domestic dogs, proper disposal of infected offal, and strict regulation of livestock slaughter. Public education in rural and pastoral communities is also vital to promote hygiene, safe slaughtering practices, and awareness of disease transmission. Veterinary and animal health services also play a central role in surveillance and prevention. Routine postmortem inspection of livestock at slaughterhouses helps detect hydatid cysts and informs disease mapping. However, in many endemic countries, informal or unsupervised slaughter remains a challenge, leading to increased environmental contamination [26].

The significance of this case lies in the necessity for medical professionals to consider hydatid disease in the differential diagnosis of cervical cystic masses, particularly in endemic regions. There, cervical hydatid disease is more likely when more common differential diagnoses fail to explain the clinical and radiological findings fully. Therefore, early diagnosis, surgical intervention, and antiparasitic therapy are crucial in preventing complications, including dissemination, anaphylaxis, and recurrence.

## 4. Conclusion

Isolated cervical hydatid cysts are extremely rare, particularly in pediatric patients, yet they should be considered in the differential diagnosis of any cervical mass, especially in endemic regions. Differentiating hydatid cysts from other congenital or benign cystic neck lesions remains a diagnostic challenge. Although imaging techniques are valuable in detecting cystic formations, they may not always reveal the exact nature of the lesion. Early diagnosis and appropriate intervention are essential in preventing complications and reducing the recurrence rate in affected individuals. Emphasis should also be placed on preventive strategies, including community-based health education on hygiene and food safety, particularly in rural and endemic areas, to help break the parasitic transmission cycle.

## Figures and Tables

**Table 1:** Summary of the WHO-IWGE classification of hydatid cysts.

Stage	Ultrasound appearance	Cyst activity	Description	Management implication
CL	Simple, unilocular cyst without specific features	Indeterminate	Cystic lesion of uncertain nature	Further evaluation needed
CE1	Unilocular, anechoic cyst with visible cyst wall	Active	Early, viable hydatid cyst	Surgery or albendazole ± PAIR
CE2	Multivesicular/multiseptated (honeycomb appearance)	Active	Advanced viable cyst with daughter cysts	Surgery or albendazole ± PAIR
CE3a	Cyst with detached membrane (“water lily sign”)	Transitional	Beginning of degeneration	Albendazole ± PAIR or close monitoring
CE3b	Solid matrix with daughter cysts	Transitional	Mixed activity; may still be viable	Usually surgery
CE4	Heterogeneous content, no daughter cysts	Inactive	Degenerating, nonviable cyst	Observation (no active treatment)
CE5	Thick, calcified wall with no fluid	Inactive	Dead, fully calcified cyst	Observation (unless complications arise)

**Table 2:** Summary of the differential diagnosis of a posterior cystic neck mass.

Condition	Etiology/pathophysiology	Clinical features	Imaging findings
Cystic lymphangioma (lymphatic malformation)	Congenital malformation of lymphatic vessels	Soft, asymptomatic, slow-growing mass; may enlarge due to hemorrhage, trauma, or infection	Multiloculated cyst with thin walls on ultrasound, CT, and MRI; minimal or absent Doppler flow
Papillary thyroid carcinoma (cystic metastasis)	Malignant lymph node involvement from thyroid cancer	Often in adolescent females; may be initial presentation of thyroid carcinoma	Cystic lymph node with thick or thin walls; hypervascular septa; calcifications or hemorrhage possible
Third branchial arch cyst	Remnant of embryonic branchial apparatus	Unilateral, painless, fluctuant mass; may become infected	Unilocular cyst; thin-walled, well-defined lesion on ultrasound or CT
Second branchial cleft cyst	Embryologic remnant of branchial cleft	Painless, fluctuant mass; located along the anterior border of the SCM muscle (can extend into the posterior neck triangle)	Well-circumscribed cyst; typically unilocular; may contain debris if infected
Neurofibroma (cystic degeneration)	Tumor of schwann cells (often in NF1)	Soft, slow-growing, nontender mass	Well-defined lesion with areas of cystic degeneration on MRI
Lipoma (cystic degeneration)	Benign adipose tumor	Soft, mobile, painless mass	Hyperechoic on ultrasound; nonenhancing on CT
Suppurative lymphadenitis (abscess formation)	Infection-related lymph node inflammation	Painful, erythematous, tender mass; systemic symptoms	Hypoechoic lymph node with central necrosis; rim enhancement on CT
Hydatid cyst (echinococcosis)	Parasitic infection (*Echinococcus* spp.)	May be asymptomatic or cause compressive symptoms	Uni or multiloculated cyst with daughter cysts; hydatid sand on ultrasound
